# Phase-Change-Material-Based True Time-Delay System

**DOI:** 10.3390/s24237613

**Published:** 2024-11-28

**Authors:** Rahuldas Kutteeri, Martino De Carlo, Francesco De Leonardis, Richard A. Soref, Vittorio M. N. Passaro

**Affiliations:** 1 Photonics Research Group, Department of Electrical and Information Engineering, Politecnico di Bari, 70126 Bari, Italy; r.kutteeri@phd.poliba.it (R.K.); francesco.deleonardis@poliba.it (F.D.L.); vittorio.passaro@poliba.it (V.M.N.P.); 2 Department of Engineering, University of Massachusetts, Boston, MA 02125, USA; richard.soref@umb.edu

**Keywords:** Bragg grating resonators, phase-changing materials, true time delay, microwave photonics

## Abstract

This study explores the achievement of a tunable true time-delay (TTD) system for a microwave phased-array antenna (MPAA) by incorporating the reversible phase-transition property of phase-change material (PCM) with Bragg gratings (BGs) and a cascade of three phase-shifted Bragg grating resonators (CPSBGRs). The goal was to design a low-power-consuming, non-volatile highly tunable compact TTD system for beam steering. A programmable on/off reflector was designed by changing a PCM-incorporated BG/CPSBGR from one phase to another. By arranging several programmable on/off reflectors in a row, a delay line was realized, and by incorporating several delay lines, the TTD system was achieved. Numerical simulations and parametric analyses were conducted for the evaluation of the TTD system’s performance at an operating wavelength of 1550 nm and 1550.6 nm for programmable on/off reflectors with BGs and CPSBGRs. The findings point out the effectiveness of incorporating PCMs with BGs/CPSBGRs, thereby maintaining a high performance with less complexity.

## 1. Introduction

The optical control of microwave phased-array antennas (MPAAs) has received attention in the past quarter of a century [1,2,3,4,5]. Unlike electrons, photons cannot be stored inside a medium; hence, most optical buffers are based on tunable true time delays (TTDs). The beam-squinting problem experienced by conventional electronic phase-array antennas (PAAs) [6] was solved using an optical TTD. An optical TTD is achieved by changing the optical path, which can be attained either using material properties such as material dispersion or by changing the length of the waveguide [7,8]. TTDs possess different applications, such as optical beamforming and beam steering, optical time-division multiplexing, microwave photonic filters, optical coherence tomography, etc. [9,10,11,12,13,14].

Previous TTD systems were based on bulk optics and fiber components. These were later replaced by on-chip TTDs as they are cost-effective, lightweight, and compact; they also provide higher operational speeds and low power consumption [15,16]. Apart from the above advantages, these on-chip TTDs can easily be combined with other passive and active devices on a chip.

Our study focused on the design and simulation work of a non-volatile optical TTD system, a more reliable method of utilizing the reversible material phase-transition properties of phase-change materials (PCMs), incorporating Bragg gratings (BGs) and a cascade of three phase-shifted BG resonators (CPSBGRs). This TTD system could be used for the beam steering of an MPAA. A change in the refractive index of PCM-incorporated BGs and CPSBGRs when changing from one material phase to another causes a difference in the optical path length of the signal, and this change in optical path length (delay) can be tuned to obtain the desired beam steering. The architecture employed in this study was inspired by a previous study [17], but with the significant replacement of a thermo-optic (TO) heater with a PCM layer to produce a better, non-volatile, and less complex TTD.

This paper is organized as follows. The background discussion explains the working principle of PCM-based silicon-on-insulator (SOI) BGs/CPSBGRs and PCM characteristics. Then, in the Numerical Results section, we present the results of the simulations carried out using PCM-based BGs/CPSBGRs for the TTD system as well as the delay-line characteristics and design. Lastly, the Discussion and Conclusions are summarized, respectively.

## 2. Background Concept

**Figure 1:**
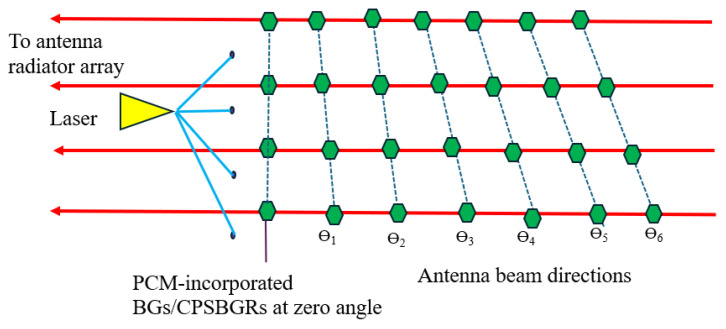
On-chip integrated optical TTD beamforming system within an antenna, which can be transformed for transmission or reception by adding circulators and waveguide circuits (modulators and amplifiers are excluded). Programmable on/off reflectors are depicted as green hexagons and optical waveguides as red lines.

Figure 1 showcases an on-chip integrated optical TTD realized by means of a set of parallel delay lines. A laser source inputs light into each of the delay lines using directional couplers [17].

Each delay line consists of several programmable on/off reflectors (depicted as green hexagons in Figure 1). Such programmable on/off reflectors are realized as PCM-incorporated BGs or CPSBGRs. Then, each delay line induces a particular delay in the reflection path according to the position of the selected on/off reflector programmed to work as a reflector.

We particularly explored antimony trisulfide (Sb_2_S_3_) as an efficient PCM. It has low intrinsic losses in both amorphous and crystalline states, showing a refractive index contrast of ∼0.60 between crystalline (3.308) and amorphous (2.712) states [18]. The temperature for crystallization of Sb_2_S_3_ is around 573 K and the melting point (required to achieve the amorphization) is around 823 K [19]. The desired wavelength of operation (∼1550 nm) was chosen as the Bragg resonance condition λ_B_ = 2n_eff_ Λ, where Λ is the grating period and n_eff_ is the effective index. The average effective index of the BG/CPSBGR could be altered in a non-volatile way by incorporating the PCM. When a particular BG/CPSBGR in a delay line is turned on (changing either from crystalline to amorphous or from amorphous to crystalline, as will be specifically indicated in the subsequent section), a remarkable shift can be observed in the reflection/transmission spectrum of the BG/CPSBGR.

**Figure 2:**
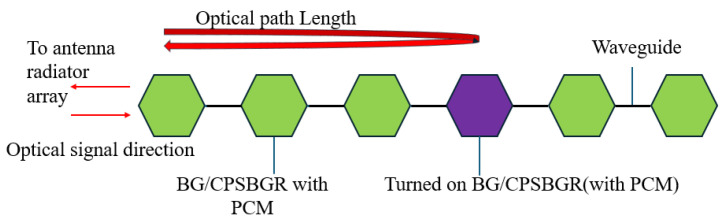
Delay line containing a series of BGs/CPSBGRs (represented as green hexagons) connected by waveguides; the violet hexagon designates the turned-on BG/PSBGR.

Initially, all PCMs in BGs/CPSBGRs are turned to an off state, with BGs/CPSBGRs already designed to work as reflectors at the operating wavelength of 1550/1550.6 nm when they are turned to an on state. Depending on the position of the BG/CPSBGR that is turned on (Figure 2) in a delay line, an optical delay is introduced due to the change in path length.

By setting up different delay lines and turning on specific programmable reflectors at different positions, desired time delays can be achieved in different delay lines. The obtained delayed signals can be utilized to modulate radio signals in an antenna radiator array; hence, it can transform into an MPAA that is able to steer the microwave beam along desired angles according to the relative delay between consecutive delay lines. All optical components are integrated in a monolithic way to obtain high compactness and efficiency.

## 3. Delay-Line Architecture

There are two methods to approach the architecture in Figure 1, meaning that the optical path selection can be achieved in two different ways, depending on the use of BGs (class I) or CPSBGRs (class II), as discussed below. In both cases, λ_0_ is the operating wavelength of the laser source. The length of the waveguide between each BG/CSPBGR is non-uniformly chosen (as will be explained in detail in the Numerical Results section). The waveguide is realized as a silicon wire immersed in SiO_2_ with a width of 450 nm and a height of 220 nm. The waveguide has an effective index, n_effw0_, of 2.347 and a group index, n_wg_, of 4.272 for both configurations.

A graphene microheater can be used by applying a voltage across it to heat the PCM in a few microseconds with high efficiency for both the BG and CSPBGR [20]. Through controlled heating and cooling, turning on specific BG/CSPBGRs can be performed.

### 3.1. Bragg Gratings

Here, the TTD system was designed to work at the operating wavelength of λ_0_ = 1550 nm. BGs work as programmable on-chip turned-on reflectors as the PCM changes from an amorphous state to a crystalline state.

**Figure 3:**
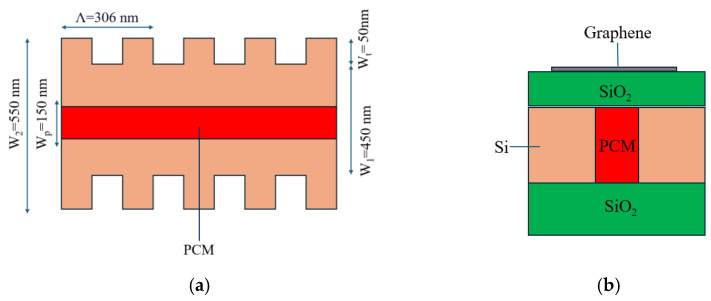
(**a**) Top view of SOI strip showing the geometry of one PCM-coated BG. (**b**) Cross-section of a SOI PCM-coated BG with a graphene microheater.

A BG was designed in such a way that it contained two alternating sections with two different widths of 450 nm and 550 nm (W_1_ and W_2_, respectively), both having a height of 220 nm (H), as shown in Figure 3a. A cross-section of a SOI PCM-coated BG with a graphene microheater is shown in Figure 3b. The period of the BG (Λ) was fixed at 306 nm to obtain a resonance wavelength of ~1550 nm in its crystalline phase; we chose 200 periods, which gave a total length of the BG of 61.2 µm. The height of the PCM was the same as that of H and the width (W_p_) was 150 nm (Figure 3a,b).

**Figure 4:**
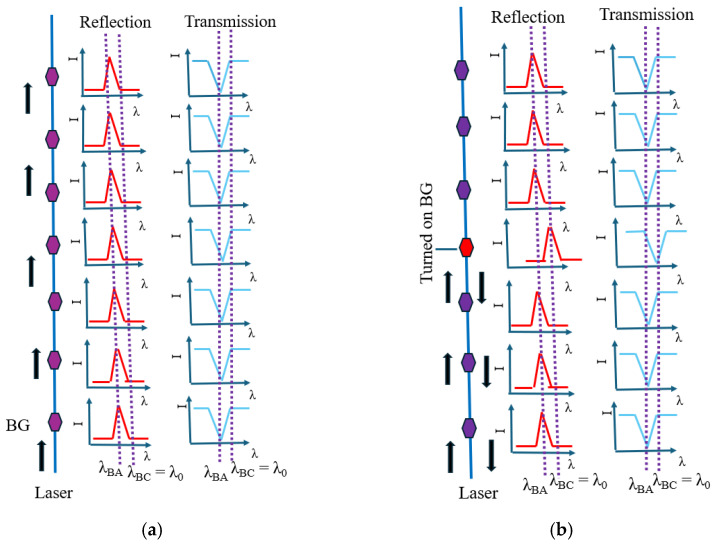
Programmable optical delay line using BGs for the selection of any one of three optical delays. Here, λ_BC_ ~1550 nm is the wavelength of operation (λ_0_). (**a**) Initial all-amorphous (λ_BA_ ~1438) BG line, with corresponding R and T profiles. (**b**) Turning on the 4th BG (amorphous to crystalline), with a shift in the R profile and a shift in the T profile for the turned-on BG.

**Figure 5:**
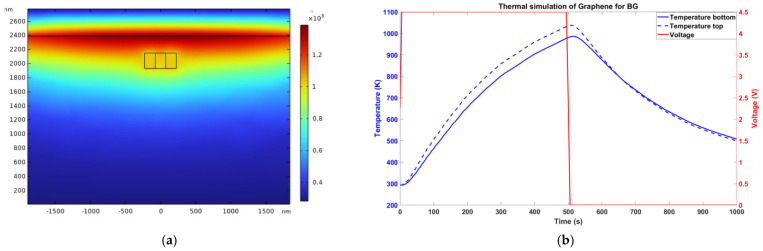
(**a**) Thermal profile (temperature in Kelvin) of the cross-section of the BG after 500 ns of a 4.5 V pulse was applied to the graphene electrodes (graphene electrodes were simulated to be 4.3 µm in length). (**b**) Temperature vs. time during the amorphization process evaluated at the top (dashed line) and bottom (solid line) for PCM of the BG.

In this case, the reflection wavelength of the turned-off reflector (amorphous state) was 1438 nm (λ_BA_). Instead, the reflection wavelength of the turned-on reflector (crystalline state) was λ_BC_, which was designed to be equal to the wavelength of operation (λ_0_). Primarily, when all the BGs in a delay line were in an amorphous state, no reflection was observed (Figure 4a). When a particular BG at a specific position was turned on, the delay line showed reflection. Thus, depending on the position of the BG turned on, a specific delay was introduced into the reflected signal of the delay line (Figure 4b). As the wavelength of operation lay in the reflection peak, this device could be named as a peak-in-reflection (or a notch-in-transmission). The thermal simulation profile corresponding with the case of the BG and the temperature as a function of time (for an applied voltage pulse of 4.5 V) are shown in Figure 5a and Figure 5b, respectively.

### 3.2. Cascade of Bragg Gratings

The TTD system, designed to work at the operating wavelength of λ_0_ = 1550.6 nm, could also be based on the notch reflection of a CPSBGR (with the same period length as the BG). The CPSGBR was designed by adding a layer of PCM (separated by 70 nm of SiO_2_) atop a Si-based phase-shifted Bragg grating resonator (PSBGR).

The CPSBGR was designed in such a way that the transmission wavelengths of the CPSBGR in a crystalline state would overlap the reflection wavelengths of the CPSBGR in an amorphous state.

**Figure 6:**
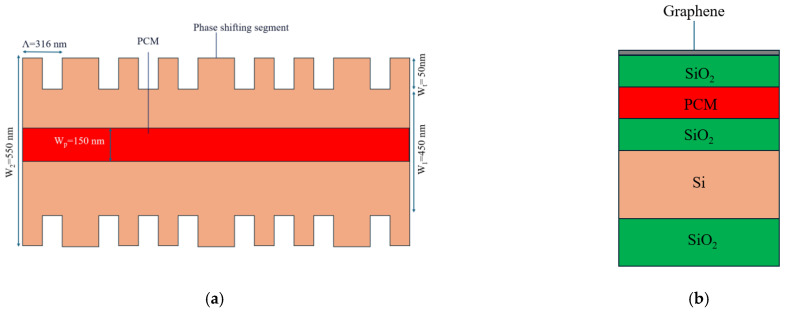
(**a**) Top view of an SOI strip showing the geometry of one PCM-coated CPSBGR. (**b**) Cross-section of a SOI PCM-coated CPSBGR including the graphene microheater.

One PSBGR in the CPSBGR had almost the same dimensions as the class Ⅰ BG, with a different period of 316 nm used to obtain a resonance wavelength of ∼1550 nm in its crystalline phase. The height of the PCM was set to 70 nm and the width (W_p_) was 150 nm. Instead of using a single PSBGR, we opted to use a cascade of three PSBGRs, named CPSBGRs (Figure 6a), and a cross-section of a SOI PCM-coated CPSBGR (including the graphene microheater) is shown in Figure 6b. The length of each phase-shifting segment was chosen to be same length as the period length, i.e., 316 nm. The total length of each CPSBGR in a delay line was chosen to be 126.4 µm to obtain the Butterworth transmittivity response, as discussed in [17,20], which provided the desired flat spectral response.

**Figure 7:**
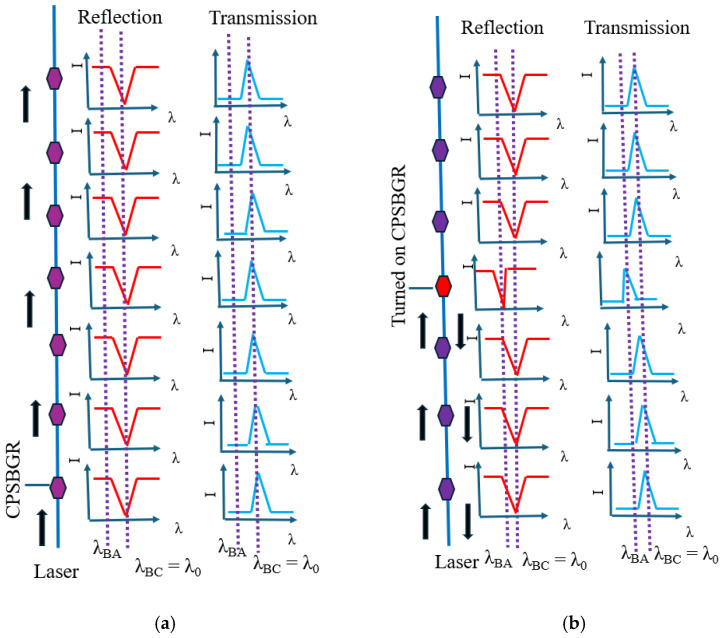
Programmable optical delay line using CPSBGRs for the selection of any one of three optical delays. Here, (λ_BC_ ~1550.6 nm) is the wavelength of operation (λ_0_). (**a**) Initial all-crystalline (λ_BC_ ~1550.6 nm) CPSBGR line, with corresponding R and T profiles. (**b**) Turning on the 4th CPSBGR (crystalline to amorphous, i.e., λ_BC_ ~1550.6 nm to λ_BA_ ~1547 nm), with a shift in the R profile and a shift in the T profile for the turned-on CPSBGR.

Initially, all CPSBGRs were kept turned off; in class II, the turned-off state referred to keeping all CPSBGRs in a crystalline state. In the turned-off state, the CPSBGRs were designed to transmit at λ_BC_ (Figure 7a). Under this condition, no signal was detected at the output because the operating wavelength λ_0_ was λ_BC_ (~1550.6 nm). Shifting of the reflection notch occurred due to the change in the refractive index as the CPSBGR at a specific position was turned on (amorphous state). In this case, the notch in the reflection spectrum shifted away from λ_BC_ to λ_BA_ (1547 nm) and the CPSBGR reflected light at the operating wavelength, λ_0_ (Figure 7b).

**Figure 8:**
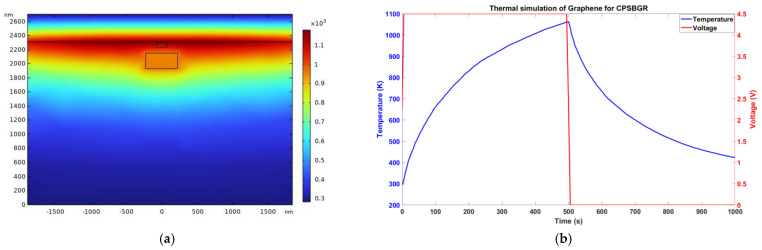
(**a**) Thermal profile (temperature in Kelvin) of the cross-section of the CSPBGR after 495 ns of a 4.5 V pulse was applied to the graphene electrodes (graphene electrodes were simulated to be 4.3 µm in length). (**b**) Temperature vs. time during the amorphization process evaluated for PCM of the CSPBGR.

Thus, a delay in class II was introduced by turning the CPSBGR from a crystalline to an amorphous state. The time delay depended on the position of the turned-on CPSBGR. As the wavelength of operation lay in the reflection notch, this device could be named as notch-in-reflection (or peak-in-transmission). The thermal simulation profile corresponding with the CSPBGR and the temperature as a function of time (for an applied voltage pulse of 4.5 V) are shown in Figure 8a and Figure 8b, respectively.

## 4. Numerical Results

**Figure 9:**
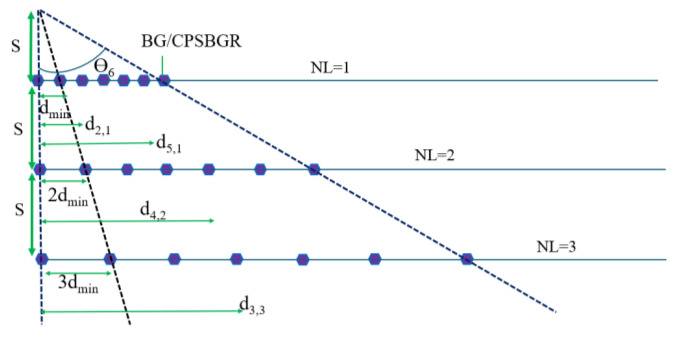
Array of delay lines with corresponding steering angles.

**Figure 10:**
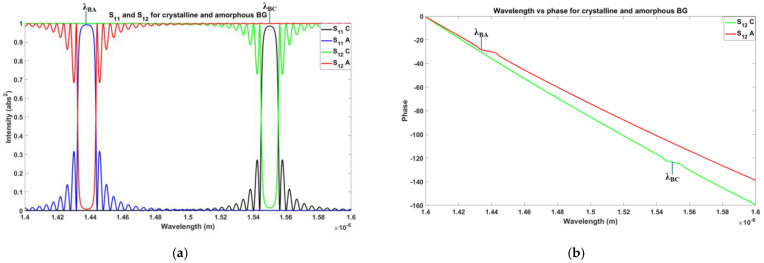
(**a**) Reflection and transmission spectral responses of PCM-coated Bragg resonator. S_11_ and S_12_ refer to reflection and transmission, respectively, in crystalline or amorphous states. (**b**) Phase dependence of the wavelength of a single BG plotted for transmission only.

**Figure 11:**
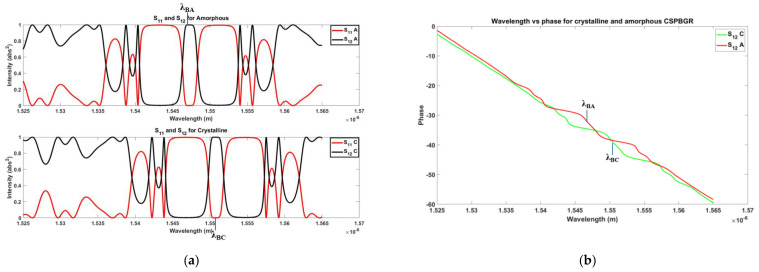
(**a**) Reflection and transmission spectral response of PCM-coated CPSBGR. S_11_ and S_12_ refer to the reflection and transmission in crystalline or amorphous states, respectively. (**b**) Phase dependence of the wavelength of a single CSPBGR plotted for transmission only.

In this Section we have investigated an array of delay lines as sketched in Figure 9. Firstly, we have analyzed the spectral profile of the designed single BG and CPSBGR, as shown in Figure 10a and Figure 11a, respectively. The shift along the wavelength axis was clearly visible in the spectral profile. Figure 10b and Figure 11b represent the phase-relation of a single BG and a single CSPBGR with the wavelength, respectively. Subsequently, we investigated a series of delay lines with seven BGs/CPSBGRs to perform beam steering (Figure 9), focusing on the X band (8 to 12 GHz) for the modulating signal. The length of the waveguide was non-uniformly chosen according to Equation (1), in which *NL* is the delay-line number and d*_min_* is the distance between the center of the first and second BG/CPSBGR along the delay line. This distance was calculated according to Equation (2), in which Ɵ*_min_* is the minimum steering angle (designated as 8°), n*_eff_* is the effective refractive index of the waveguide, *f_max_* is the maximum microwave frequency in the band, and c is the speed of light in a vacuum. The design was adapted for a few angles, Ɵ*_i_*, ranging from 8° to 16°, 24°, 32°, 40°, or 48° [17]. In this work, the simulation and numerical calculations were carried out by an eigenmode expansion (EME) analysis [21] using the MODE module of Lumerical software (version 1.9.3724) [22] and the Transfer Matrix approach [23]. An integrating framework was applied for the full vectorial modelling of optical propagation in photonics structures such as BGs/CSPBGRs as well as the waveguide.

(1) $$d_{i,NL}=\left(NL\right)d_{min}\left(\frac{\tan{Ɵ}_{i}}{\tan{Ɵ}_{min}}\right)$$

(2) $$d_{min}=c\left(\sin{Ɵ}_{min}\right)/\left(4f_{max}n_{eff}\right)$$

The vertical distance between the two delay lines (*S*) in Figure 11 was calculated to be 1.25 cm according to Equation (3), where λ(min) is the minimum microwave band wavelength for the X band (8–12 GHz).

(3) $$S=0.5\times \lambda \left(\min\right)$$

The minimum distance in the case of the BG and CSPBGR was the same as it only depended on the effective index of the waveguide, steering angle, and maximum microwave band frequency. The calculated d*_min_* using Equation (2) was 370.6 µm.

The lengths of the non-uniformly chosen waveguides in between the BG/CPSBGR in the first delay line for different angles are given in Table 1.

**Figure 12:**
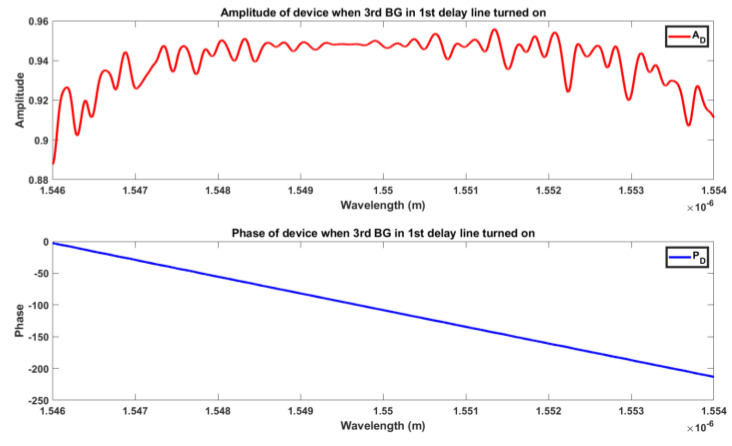
Amplitude (A_D_) and phase (P_D_) of the device when the 3rd BG in the first delay line was turned on.

**Figure 13:**
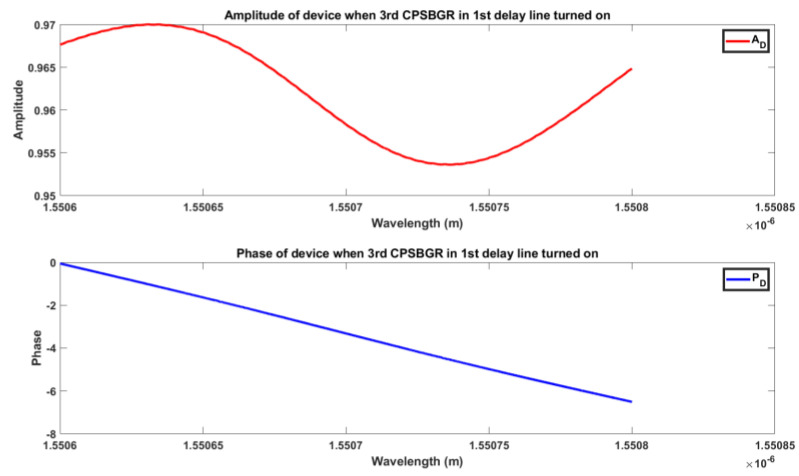
Amplitude (A_D_) and phase (P_D_) of the device when the 3rd CPSBGR in the first delay line was turned on.

**Figure 14:**
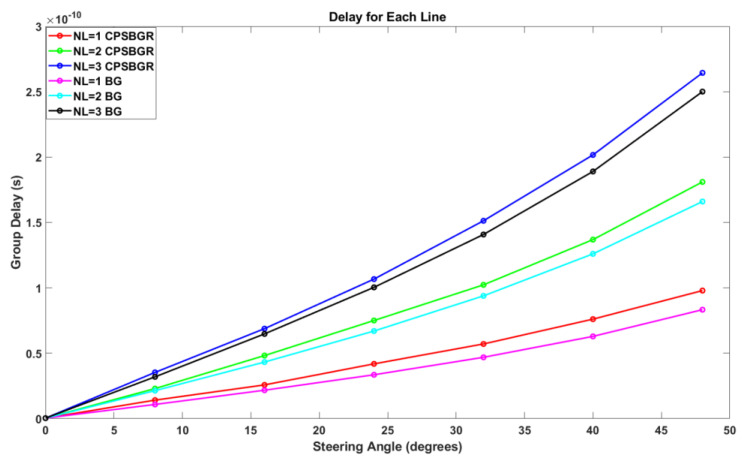
Group delay induced when each BG/CPSBGR was turned on for different steering angles and different delay lines (NL).

Figure 12 and Figure 13 illustrate an example of the overall amplitude and phase of a designed delay line when the third BG or CPSBGR was turned on, respectively. Figure 14 shows the different time delays induced when each BG was turned on for different delay lines and for steering at different angles such as 8° to 16°, 24°, 32°, 40°, or 48°. The maximum induced time delays (i.e., when the last BG was turned on) for each line were 83.3 ps, 166 ps, and 250 ps, respectively, and the maximum time delays induced for the CPSBGR across different delay lines were 97.9 ps, 181.6 ps, and 264.4 ps, respectively. The relative delay between different delay lines gave the desired steering angle.

## 5. Discussion

The tuning of time delay was achieved by delay lines containing BGs/CPSBGRs. The time delay increased with the position number of the turned-on BGs/CPSBGRs in a specific delay line. Beam steering for the desired angle could be achieved by the incorporation of several delay lines and by turning on specific BGs/CPSBGRs as reflectors. In the delay lines, the separation between the first and second BG/CPSBGR was chosen as the integer multiple of d_min._ Beam steering was achieved by the collective performance of programmable on/off reflectors (BGs or CPSBGRs) in different delay lines. This study provides insights into the advantage of PCM integration in BGs/CPSBGRs, demonstrating the feasibility of digital programming for beamforming in microwave phased-array antennas. This could be used for the modulation of the light phase and amplitude. The results of the study open various new possibilities and applications in communication systems through the integration of PCMs with photonic devices and, subsequently, for the further development of advanced integrated photonics.

## 6. Conclusions

This work mainly focused on how the incorporation of a phase-change material with a Bragg grating and a cascade of three phase-shifted Bragg grating resonators could be utilized to obtain a tunable true time delay in integrated phase-array antennas. Due to the reversible material phase-transition properties and refractive index contrast between the material phases of the phase-change material, programmable non-volatile on/off Bragg grating/cascade of three phase-shifted Bragg grating was simulated, and beam steering could be achieved at ~1550 nm and ~1550.6 nm using these programmable on/off devices. A high-performing true time-delay system, compared with the previously studied true time-delay system that used thermo-optic switching, was achieved by replacing thermo-optic switching with the reversible phase-transition property of the phase-change material and by incorporating it with Bragg grating and a cascade of three phase-shifted Bragg grating. This work poses certain advantages compared with prior work, especially in relation to power efficiency. As PCMs have a non-volatile nature, they do not need a continuous power supply to hold delay settings; hence, non-volatile beam steering can be achieved whereas in prior work, thermo-optic tuning required ongoing power to maintain the desired delay. Also, the large refractive index induced by the phase-change material reduced the system dimensions. This opens up various research opportunities in telecommunications.

## Figures and Tables

**Table 1:** d_i,NL_ values and corresponding steering angles for NL = 1.

Steering Angle (Degree)	d_i,NL_ (µm) BG/CSPBGR
8	370.6
16	756.1
24	1200
32	1600
40	2200
48	2900

## Data Availability

Research data for this study can be available upon reasonable request.
